# Validität und Reliabilität virtualisierter RTR-Messungen

**DOI:** 10.1007/s11616-021-00680-1

**Published:** 2021-07-26

**Authors:** Thomas Waldvogel, Uwe Wagschal, Thomas Metz, Bernd Becker, Linus Feiten, Samuel Weishaupt

**Affiliations:** 1 Seminar für Wissenschaftliche Politik, Professur für Vergleichende Regierungslehre, Albert-Ludwigs-Universität Freiburg, Werthmannstraße 12, 79098 Freiburg, Deutschland; 2 Albert-Ludwigs-Universität Freiburg, Freiburg, Deutschland

**Keywords:** Real-Time Response Measurement, RTR, Reliabilität, Validität, TV-Duelle, Real-Time Response Measurement, RTR, Reliability, Validity, Televised debates

## Abstract

Können virtualisierte Erhebungen rezeptionsbegleitend gemessener Kandidatenbewertungen in Echtzeit über das Internet valide und reliable Daten generieren? Seitdem Reinemann et al. vor 15 Jahren die Reliabilität und Validität von sogenannten Real-Time-Response-Messungen (RTR) mit physischen Eingabegeräten in laborexperimentellen Erhebungssettings in dieser Zeitschrift belegt haben, hat die Messtechnik eine weitreichende Veränderung erfahren, die durch die Virtualisierung ihres Instrumentariums gekennzeichnet ist. Allerdings liegen bislang kaum methodologische Erkenntnisse über den grundlegenden Wandel dieser Schlüsseltechnik vor. Der hier vorliegende Beitrag untersucht deshalb anhand von drei Fernsehduellen zu Landtagswahlen in Deutschland, inwiefern Daten, die mit einem virtualisierten Messinstrument der RTR-Messung in einer Feldstudie erhoben wurden, etablierten Standards der Datenqualität entsprechen. In unserer Analyse finden wir deutliche Belege für die Validität des Messverfahrens. Darüber hinaus zeigt unser Beitrag, dass diese neuartige Form der Erhebung von Echtzeitreaktionen reliable Daten mit einer hohen internen Konsistenz generieren kann, wenngleich die Befunde in Teilen ambivalent bleiben. Wir schlussfolgern, dass die Virtualisierung des RTR-Messinstrumentariums einen komplementären Ansatz zu den dominierenden Erhebungen mit laborexperimentellen Forschungsdesigns etabliert, wodurch die Analyse von Publikumsreaktionen auf landespolitische TV-Duelle in natürlichen Rezeptionssituationen zugänglich und die technische Implementierung von RTR-Studien über das Internet auch in Zeiten der COVID-19-Pandemie gewährleistet wird.

## Abstract (en)

Real-Time Response Measurement (RTR) is an important approach in political communication research to investigate human processing of political information. Since Reinemann et al. demonstrated the reliability and validity of RTR with physical input devices in laboratory experimental study designs in this journal 15 years ago, measurement technology has experienced an instrumental change characterized by its virtualization. At the same time, there still is little methodological knowledge available about the fundamental changes in this key technology. Therefore, this article examines the extent to which data collected in a field study using a virtualized RTR-Measurement tool meet established standards of data quality.

Therefore, our first research question examines the validity of the RTR data in three ways. First, we consider construct validity. The results of our Kruskal-Wallis tests show a statistically significant differentiation of RTR rating behavior along party lines. This positive assessment is underlined by the high match score of a discriminant analysis. In a further analysis step, we implement a sparse structural equation model that assesses the relationship between party identification and RTR measurement, allowing us to draw conclusions about the construct validity of the virtualized RTR measurement. The calculated coefficients provide evidence that party identification and real-time rating are significantly and substantially associated. Although our analysis focuses merely on the partisan groups of the two candidates which parties are represented in the TV debates, in summarizing the results, we consider the question of construct validity (FF1.1) to be positively decided.

As a second perspective, we investigate criterion validity. A central finding here emerges from an examination of the sparse structural equation model, at the core of which is the relationship of RTR ratings and retrospective verdicts on debate performance. Controlling for party identification, we can show that the coefficients are very strong and significant. This observation holds across all duels and candidates. We thus find a consistent pattern, which leads us to answer the question about criterion validity (FF1.2) of our virtualized RTR measure in the affirmative.

As a third perspective, we focus on content validity. For this purpose, we refer to an established structural equation model that embeds the RTR signal in a complex framework of upstream and downstream variables of debate reception. Our results are broadly consistent across duels and candidates: real-time evaluations during the debate are significantly preformed by political predispositions (prior candidate evaluation). Consistent with our previous findings, perceived debate performance is largely a function of RTR ratings in this more complex model; this underscores the previously positive assessment about the criterion validity of the virtualized RTR measure. In turn, the retrospective candidate evaluations are significantly shaped by the real-time ratings and the retrospective verdicts on debate performance. Consequently, the framework used shows that the structure of debate perception in our field studies corresponds to findings known from studies in laboratory settings with physical input devices. We are therefore confident to answer the question of content validity (FF1.3) of our virtualized RTR measurement positively.

Our second research question focuses on the reliability of the RTR data and examines it in two ways. First, using a resampling approach, we take a close look at the aggregated RTR time series. On the one hand, we find that virtualized RTR measures can indeed generate data whose aggregate RTR time series for two subsamples are clearly associated with each other in the resampling procedure. On the other hand, the findings are ambivalent because the existing correlations are only of moderate strength and in some cases only weak. Thus, the RTR measure seems to depend on the composition of the sample to a considerable extent. In a second analysis strategy, we check the individual RTR time series for internal consistency. Both Cronbach’s alpha and McDonald’s omega demonstrate a high degree of intercorrelation between the RTR ratings of the individual speech phases. When summarizing our findings on the aggregated and individual RTR time series, we must acknowledge limitations to our research question two (FF2): Virtualized RTR measurements can generate reliable data. However, this is not an automatism; rather, researchers must take special caution to ensure the conditions for an adequate study design in the online setting.

Acknowledging that our results are subject to multiple limitations, we conclude that the virtualization of RTR-Measurement establishes a complementary approach to studies with a laboratory research design dominating the literature, making the analysis of viewers’ reactions to televised debates in natural reception situations accessible even in times of the COVID-19 pandemic.

## Einleitung

Im Fernsehen übertragene Diskussionen zwischen den Spitzenvertretern politischer Parteien vor Wahlen können als Kulminationspunkt politischer Kommunikation gelten. Für die kommunikations- und politikwissenschaftliche Analyse bieten sie ein besonders erkenntnisreiches Forschungsfeld, weil sie die exemplarische und klar abgrenzbare Untersuchung eines Forschungsobjektes erlauben, dessen Erkenntnisse über die konkrete Kommunikationssituation hinausreichen (vgl. Vögele et al. 2013, S. 31). In der empirischen Debattenforschung sind dabei Experimente mit Vorher-Nachher-Messung weit verbreitet, ebenso wie Bevölkerungsbefragungen (vgl. Reinemann et al. 2005, S. 56). Ihnen ist gemein, dass sie den Medieninhalt als Einheit betrachten und folglich keine näheren Aussagen über die Informationsverarbeitung von Rezipienten während der Stimulusrezeption erlauben. Als besonders erkenntnisreich erweisen sich deshalb Studien, die die Messung von Echtzeitreaktionen der Zuschauer während der Stimulusrezeption in das methodische Zentrum der Analyse stellen. Diese sogenannten Real-Time-Response-Messungen (RTR) ermöglichen es den Probanden, ihre spontanen Eindrücke über eine Debatte unmittelbar mit Hilfe eines Eingabegerätes sekundengenau mitzuteilen. Diese Eingaben werden mit einem Zeitstempel und Pseudonym versehen und zentral gespeichert. Forschende haben dann die Möglichkeit, diese Daten grafisch aufzubereiten und statistisch auszuwerten. Die Messung der Echtzeitreaktionen wird für gewöhnlich mit einer zumindest zweiwelligen Befragung vor und nach der Rezeption verbunden (vgl. Biocca et al. 1994; Ottler 2013; Waldvogel und Metz 2017). RTR-basierte Studien zu politischen TV-Debatten konnten zeigen, dass motivationale Variablen wie das Interesse am Wahlkampf und die Partizipationsbereitschaft (vgl. Faas und Maier 2004a; Maier et al. 2013; Range 2017), kognitive Größen wie das Urteilsvermögen, politisches Wissen und das subjektive Kompetenzgefühl (vgl. Faas und Maier 2011; Maier 2007a; Maier et al. 2013; Maurer und Reinemann 2006), kandidatenbezogene Einstellungen wie die Kandidatenpräferenzen und -images (vgl. Maier und Faas 2003; Maurer und Reinemann 2003; Maurer et al. 2007; Bachl 2013; Maier 2007b; Waldvogel 2019) oder verhaltensrelevante Aspekte wie die Wahlabsicht (vgl. Faas und Maier 2004b; Maier, M. 2007; Maier et al. 2013) von der TV-Duell-Rezeption maßgeblich beeinflusst werden.

Während sich seit der Etablierung politischer TV-Debatten in Deutschland also eine ausdifferenzierte Forschungslandschaft entwickelt hat, die detaillierte Befunde über die Inhalte, Wahrnehmungen und Wirkungen beförderte, fanden methodologische Ansätze, die die Qualität der erhobenen Echtzeitdaten, deren Reliabilität und Validität fundiert in den Blick nehmen, weit weniger Beachtung (vgl. Maier et al. 2009, 2007; Reinemann et al. 2005). Dies erscheint insbesondere deshalb unzureichend, weil sich die RTR-Messung seit wenigen Jahren in einem tiefgreifenden Wandel befindet, der sich in der Virtualisierung ihres Messinstrumentariums vollzieht (vgl. Maier et al. 2016; Metz et al. 2016; Waldvogel und Metz 2020). Kernidee ist es, die Erhebung von Echtzeitdaten in Feldstudien zu ermöglichen, in denen Studienteilnehmer ihre allgemeinen Urteile und unmittelbaren Wahrnehmungen über eine TV-Diskussion mithilfe eines mobilen Endgerätes über das Internet in natürlichen Rezeptionssituationen mitteilen können – zum Beispiel vor dem heimischen Fernsehgerät. Mit diesem mobilen Ansatz scheinen Vorteile verknüpft: Die Hürde vor der Teilnahme an einer Studie (z. B. die Anfahrt ins Labor) wird verringert, was die räumliche Repräsentation der Stichprobe verbessern kann, da die Teilnehmer kein Labor mehr betreten müssen und von zu Hause aus über das Internet auf den Medienreiz in natürlichen Rezeptionssettings reagieren können. Zweitens werden die Kosten für das Messinstrumentarium und somit für die Studie insgesamt reduziert, da Probanden die eigene Hardware nutzen. Drittens wird die Flexibilität des RTR-Instruments erhöht, da verschiedene Konfigurationen der grafischen Benutzeroberfläche (GUI) softwarebasiert implementiert werden können (vgl. Waldvogel 2020a, S. 24–25).

Wenngleich dieser Ansatz also forschungspraktische Vorteile verspricht, so ziehen die (gegenüber einer Laborsituation) geringeren Standardisierungs- und Kontrollmöglichkeiten der Stimulusrezeption die Qualität virtualisiert erhobener RTR-Daten in Zweifel. Dies kann tiefgreifende Auswirkungen auf die inhaltliche Interpretationsfähigkeit von RTR-Eingaben haben, wenn diese nicht mehr zuverlässig mit den Kandidatenaussagen im TV-Duell korrespondieren. So ließe sich beispielsweise kaum analysieren, inwiefern es politische Prädispositionen oder vielmehr die Performanz eines politischen Wettbewerbers im TV-Duell ist, die die Kandidatenpräferenz leitet. Aktuelle Forschungsarbeiten haben dieses Problem bisher allerdings kaum beachtet. Ziel des vorliegenden Artikels ist es, einen Beitrag zur Schließung dieser Forschungslücke zu leisten und Antworten auf zwei Forschungsfragen zu geben:

1. Ist die Datenstruktur virtualisierter RTR-Messverfahren valide (FF1)?
2. Können virtualisierte RTR-Messungen reliable Daten generieren (FF2)?

Zur Beantwortung dieser beiden Fragestellungen nutzen wir Daten aus RTR-Studien zu drei Landtagswahlen (vgl. Waldvogel 2020b), indem wir die methodologischen Aspekte der empirischen Studien auskoppeln. Im Kern greift die Untersuchung auf Echtzeitdaten zurück, die mit dem „Debat-O-Meter“, einem virtualisierten Instrument der RTR-Messung, erhoben wurden. Diese werden von einer zweiwelligen Panelbefragung unmittelbar vor und nach der Rezeption der TV-Debatten zu den Landtagswahlen 2017 in Schleswig-Holstein, Nordrhein-Westfalen (NRW) und Niedersachsen gerahmt. Damit wird eine fundierte Beurteilung der Qualität von RTR-Daten, die im Rahmen von Feldstudien in natürlichen Rezeptionssituationen erhoben wurden, aus einer methodologischen Perspektive möglich.

Der Beitrag beginnt mit einem kurzen Überblick über den methodologischen Forschungsstand zur RTR-basierten Debattenforschung in Deutschland und entfaltet dabei unterschiedliche Konzeptionen von Validität und Reliabilität. In Abschn. 3 erfolgt daraus die Ableitung empirisch überprüfbarer Hypothesen. Der 4. Abschnitt beschreibt das Studiendesign, präsentiert das Debat-O-Meter als virtualisiertes Messinstrument der RTR-basierten Debattenforschung und spezifiziert die Operationalisierung. Analyseabschnitt 5 präsentiert die zentralen Befunde über die Validität und Reliabilität des virtualisierten Messverfahrens. Abschließend werden die Ergebnisse diskutiert und Schlussfolgerungen hinsichtlich der eingangs formulierten Forschungsfragen gezogen.

## Forschungsstand

### Validität

Zur Beurteilung der Validität von RTR-Daten lassen sich drei für diese Arbeit relevante Konzepte unterscheiden (vgl. Wagschal 1999, S. 39–41): Die Konstruktvalidität stellt die Frage, ob und in welchem Ausmaß ein Messkonzept mit dem zugrunde liegenden theoretischen Konstrukt korrespondiert. Unter der Annahme, dass die Echtzeitbewertungen der Zuschauer wesentlich durch die Parteienidentifikation geprägt werden, die als eine stabile und affektive Bindung einer Person an eine politische Partei die Wahrnehmung politischer Inhalte und Akteure maßgeblich vorformt (vgl. Campbell et al. 1960, S. 130), wird im Kontext von RTR-Messungen Konstruktvalidität als eine ausgeprägte Assoziation zwischen den gemessenen RTR-Bewertungen und der Parteienidentifikation definiert (vgl. Maier et al. 2007, S. 66). Zweitens wird mit der Kriteriumsvalidität die prognostische Qualität des Messkonzeptes auf ein externes Messkriterium beurteilt. Für die Rezeption politischer TV-Diskussionen dürfen wir annehmen, dass sich die unmittelbaren Eindrücke zu einem retrospektiven Gesamturteil nach der Debatte saldieren (vgl. Ottler 2013, S. 126). Es stellt sich also die Frage, inwiefern die nach der Debatte getroffenen Urteile über die Debattenleistungen der Kandidaten mit den spontanen Echtzeitreaktionen der Zuschauer während der Diskussion assoziiert sind. Drittens lenkt die Inhaltsvalidität den Blick darauf, ob das Messinstrument inhaltlich (sachlich und logisch) geeignet ist, das interessierende Merkmal zu erfassen.

Eine Vielzahl an Studien konnte wiederholt belegen, dass das erfasste RTR-Signal inhaltliche Interpretationen über die Strukturen der Wirkung und Wahrnehmung der Medienstimuli erlaubt und folglich Beziehungen zu anderen Variablen der Debattenrezeption sachlich und logisch nachvollziehbar abbildet (vgl. Maier 2007b; Maier et al. 2014). Für die politische Ebene von Landtagswahlen demonstriert beispielsweise Bachl (2013) nicht nur die Verbindung von Inhaltsanalyse und RTR-Bewertungen, sondern auch die zentrale Bedeutung der Echtzeitevaluation für das Wirkungsgeflecht zwischen vor- und nachgelagerten Variablen der Debattenrezeption (wie z. B. die Bewertung der Kandidaten). Insbesondere konnte gezeigt werden, dass die skizzierten Zusammenhänge zwischen Parteiidentifikation, RTR-Signal und Debattenleistung mit Blick auf die Konstrukt- und Kriteriumsvalidität bestehen (vgl. Bachl 2013; Maier et al. 2014; J. Maier 2007b; Papastefanou 2013). Sie zeichnen damit ein positives Bild der Validität für laborexperimentelle RTR-Messungen, die physische Eingabegeräte verwenden.

Mit Blick auf virtualisierte RTR-Messungen belegen Maier et al. (2016) nicht nur die Konstruktvalidität, indem sie die Beziehung zwischen Echtzeitreaktionen und Parteienidentifikation analysieren. Sie verifizieren auch die Kriteriumsvalidität, indem sie die Korrelationen zwischen den RTR-Bewertungen und dem wahrgenommenen Debattensieger untersuchen. Diese Korrelationen werden durch eine Pfadanalyse bestätigt, die zu einer bekannten Struktur der Debattenrezeption führen, wie sie aus früheren Untersuchungen zu Fernsehdebatten in Deutschland bekannt sind (vgl. Bachl 2013; Maier et al. 2007). Waldvogel und Metz (2020) entwickeln diesen Ansatz weiter: Auf Basis von Kruskal-Wallis-Tests mit Post-Hoc-Vergleichen zeigen sie, dass das RTR-Signal verschiedene parteipolitische Anhängergruppen diskriminiert. Dies verweist auf eine starke Verbindung zwischen Parteiidentifikation und Echtzeitbewertung und dient den Autoren als Beleg, dass Konstruktvalidität gegeben ist. Darüber hinaus zeigen ihre Strukturgleichungsmodelle, dass die RTR-Ratings signifikant und substantiell mit den retrospektiven Urteilen der Teilnehmer über die Debattenleistung assoziiert sind. Das deutet darauf hin, dass beide Aspekte der Validität auch in Feldstudien mit geringeren Möglichkeiten der Kontrolle und Standardisierung der Stimulusrezeption gegeben sein können, in denen Probanden ihre allgemeinen Einstellungen und spontanen Eindrücke über eine politische TV-Diskussion in natürlichen Situationen außerhalb des Labors über das Internet online mitteilen.

### Reliabilität

Die methodologische Forschung zeichnet für die unterschiedlichen Formen physischer Eingabegeräte wie Dreh‑, Schieberegler und Druckknopfsysteme insgesamt ein positives Bild über die Reliabilität des RTR-Messverfahrens (vgl. Hallonquist und Suchman 1944; Hallonquist und Peatman 1947; Schwerin 1940), wenngleich die Erkenntnisse immer noch fragmentiert sind (vgl. Bachl 2014, S. 53–63; Papastefanou 2013).

Neben diesen vielversprechenden Befunden zu physischen RTR-Eingabegeräten untersuchen nur wenige Studien die Reliabilität virtualisierter RTR-Messungen. Metz et al. (2016) berichten eine Korrelation von 0,77 beim Vergleich einer virtualisierten Schieberegler-Implementierung mit physischen Drehreglern für zwei randomisierte Gruppen in einem kontrollierten, quasi-laborexperimentellen Studiendesign. Diese Ergebnisse werden von Maier et al. (2016) gestützt, die in ihrem Paralleltest von zwei nicht randomisierten Gruppen Koeffizienten von über 0,51 berichten. Die Gruppen verfolgten dieselbe Debatte – eine im Labor mit physischen Eingabegeräten und eine weitere Gruppe zu Hause mit einer virtualisierten RTR-Applikation. In Erweiterung des von Papastefanou (2013) und Bachl (2014, S. 106–110) für laborbasierte RTR-Erhebungen dargelegten Ansatzes für die Beurteilung der internen Konsistenz von RTR-Daten berichten Waldvogel und Metz (2020) in ihrer Studie sowohl für Cronbachs Alpha als auch für McDonalds Omega Werte nahe oder über 0,90, was ebenfalls auf eine hohe Reliabilität virtualisierter RTR-Messungen hinweist.

## Forschungsfragen und Hypothesen

Mit der Implementierung virtualisierter Formen der RTR-Messung scheinen vielfältige Erwartungen verknüpft: Die externe Validität der Erhebung soll verbessert, die Hürden für die Studienteilnahme sollen gesenkt, die räumliche Repräsentation der Stichprobe erhöht und die Kosten für die technische Ausstattung reduziert werden (vgl. Waldvogel 2020b, S. 24–25). Gleichzeitig birgt das Verlassen des Labors aber auch ein erhebliches Risiko, da die Forscher keine Kontrolle über das Umfeld haben, in dem die Teilnehmer die Debatte verfolgen. Folglich haben sie auch keine Möglichkeit, eine Standardisierung des Erhebungsprozesses und ausreichende Datenqualität sicher zu gewährleisten. So könnten zusätzliche Quellen der Ablenkung beispielsweise durch parallele Social-Media-Aktivitäten zu inkonsistenten Bewertungsmustern in Echtzeit führen (Reliabilität) oder der unkontrollierte Konsum von Vor- und Nachberichterstattung zu einem TV-Duell die Assoziationen des RTR-Signals zu vor- und nachgelagerten Variablen der Debattenrezeption negativ beeinflussen (Validität).

Wir haben deshalb eingangs unsere erste Forschungsfrage formuliert: Ist die Datenstruktur virtualisierter RTR-Messverfahren valide (FF1)? Als Ansätze für die Analyse der Validität haben wir die drei Konzepte der Konstrukt‑, Kriteriums- und Inhaltsvalidität beschrieben (vgl. Bachl 2013; Maier et al. 2007, 2016; Wagschal 1999, S. 40). Vorherige Studien belegen die Konstruktvalidität anhand der Assoziation zwischen dem RTR-Signal und der Parteiidentifikation, was uns zu der Frage veranlasst, ob wir eine äquivalente Assoziation auch für unsere virtualisierten RTR-Daten finden können (FF1.1). Ausgehend von einer grundlegenden Freund-Feind-Logik der sozialen Identität, die im Zentrum der Parteiidentifikation steht (vgl. Green et al. 2002) sowie konsistenztheoretischen Überlegungen (vgl. Festinger 1962; Heider 1958; Faas und J. Maier 2004b), die zu selektiven Wahrnehmungen politischer Inhalt führen, vermuten wir, dass die verschiedenen Gruppen politischer Anhängerschaft in ihren Echtzeitbewertungen signifikant voneinander unterscheidbar sind (H1). Des Weiteren kann die Validität im Sinne einer Kriteriumsvalidität beurteilt werden, indem der Zusammenhang zwischen den Echtzeitreaktionen und einem externen Kriterium wie z. B. der wahrgenommenen Debattenleistung analysiert wird (FF1.2). Wir gehen dabei von der Hypothese aus, dass die Echtzeit-Evaluationen und retrospektiven Urteile über die Debattenleistung unter Kontrolle der politischen Voreinstellung der Parteiidentifikation nicht nur signifikant, sondern auch substanziell korreliert sind (H2). Mit der Inhaltsvalidität (FF1.3) weiten wir den Blick über die beschriebenen Zusammenhänge hinaus und nehmen an, dass die virtualisierte RTR-Messung einer Struktur der Debattenrezeption folgt, wie sie aus Untersuchungen mit laborexperimentellen Studiendesigns und physischen Eingabegeräten bekannt ist (vgl. Bachl 2013), wobei die unmittelbaren Wahrnehmungen in Echtzeit substanziellen Einfluss auf die retrospektiven Kandidatenbewertungen nehmen (H3).

Unser zweiter Analyseschwerpunkt liegt auf der Frage, ob virtualisierte RTR-Messungen reliable Daten generieren können (FF2). Zur Beantwortung dieser Frage nehmen wir zwei Perspektiven ein: Um die Reliabilität der *aggregierten* RTR-Zeitreihen zu beurteilen, folgen wir Bachls (2014, S. 101–106) Ansatz zur Reliabilitätsprüfung laborexperimenteller RTR-Daten und implementieren ein dem Split-Half-Design entlehntes Resampling-Verfahren. Wenn die aggregierten RTR-Zeitreihen zuverlässig Auskunft über die Bewertung der Kandidaten durch die gesamte Gruppe geben, sollten zwei Zeitreihen, die jeweils aus den Bewertungen der Hälfte der Rezipienten zufällig gebildet wurden, miteinander korreliert sein. Ist dies nicht der Fall, so wäre der Verlauf der aggregierten Zeitreihen in starkem Maße von der individuellen Zusammensetzung der Stichprobe abgängig und die Reliabilität des Messverfahrens in Frage gestellt. Auf Grund der variablen Befunde, wie sie zum Beispiel Bachl (2014, S. 60) berichtet, und weil es keinen allgemeinen Schwellenwert für die Beurteilung von Korrelationen zwischen aggregierten RTR-Zeitreihen gibt (vgl. Maier et al. 2016, S. 549), folgen wir der allgemeinen Methodenliteratur (vgl. Wagschal 1999, S. 197), indem wir Korrelationskoeffizienten von über 0,6 als mittelstark und damit ausreichend beurteilen, um eine akzeptable Reliabilität der aggregierten RTR-Zeitreihen anzuzeigen (H4).

Um die Reliabilität der *individuellen* RTR-Zeitreihen zu prüfen, können McDonalds Omega und Cronbachs Alpha als in der Literatur etablierte Indikatoren gelten (vgl. Papastefanou 2013; Bachl 2014, S. 60; Waldvogel et al. 2021). Diese Parameter messen die interne Konsistenz der RTR-Eingaben über die Zeit hinweg. Die Grundidee dieses Ansatzes ist es, die Abfolge eingehender RTR-Ratings als ein Test-Retest-Szenario zu interpretieren, bei dem das RTR-Signal als eine Längsschnittmessung betrachtet wird, in der wiederholt dasselbe Item über die gesamte Dauer der Debattenrezeption abgefragt wird. Wenn das virtualisierte Messinstrumentarium die Echtzeitreaktionen der Zuschauer zuverlässig erfasst, so sollten die Echtzeitbewertungen der Probanden zumindest in kurzfristiger Perspektive nicht stark variieren. Vielmehr werden substanzielle Interkorrelationen zwischen den Quasi-Items als Zeichen dafür gewertet, dass das virtualisierte Messverfahren als intern konsistent und damit reliabel angesehen werden kann (vgl. Waldvogel und Metz 2020). Während in der Literatur Koeffizienten von über 0,9 ein sehr hohes Maß an Reliabilität anzeigen, werden Werte über 0,8 als akzeptabel angesehen (vgl. Bortz und Döring 2006, S. 199). In der Annahme, dass unsere virtualisierten RTR-Daten reliabel sind, vermuten wir, dass die Indikatoren zur Beurteilung der individuellen RTR-Zeitreihen in unserer Studie über der entscheidenden Schwelle von 0,8 verbleiben (H5).

## Daten und Methoden

### Eingabegerät und Studiendesign

Als instrumentelle Basis zur Beurteilung der entfalteten Forschungsfragen und Hypothesen nutzen wir das sogenannte Debat-O-Meter. Die linear-modulare Struktur dieses virtualisierten RTR-Messinstrumentariums ist dem Studiendesign klassischer laborexperimenteller Erhebungen entlehnt und zielt in einer Art von „virtuellem Versuchslabor“ darauf ab, die gängige Phasenstruktur von RTR-Studiendesigns online umzusetzen: Nach einer kurzen Anleitung, in der die Bedienoberfläche erklärt und die Messanweisung (siehe Anhang A) erläutert wird, folgt eine Vorbefragung, in der verschiedene Variablen (Soziodemographie, politische Einstellungen und Verhaltensweisen, Einschätzungen und Erwartungen an die Debatte und die Diskutanten) abgefragt werden. Kern der Web-Anwendung bildet das RTR-Modul, mithilfe dessen die Nutzer ihre (der Messanweisung entsprechende) Bewertung in Echtzeit abgeben können. Diese Eingaben werden sekundengenau vom Debat-O-Meter erfasst, mit der pseudonymisierten Nutzerkennung und einem Zeitstempel versehen, bevor sie gemeinsam in der Datenbank auf einem nach Nutzerlast skalierbaren Server gespeichert werden. Am Ende der Debatte werden die Teilnehmer unmittelbar zur Nachbefragung weitergeleitet. Eine Übersicht über die in den Befragungen erhobenen und für diese Untersuchung relevanten Konstrukte ist in Anhang B dargestellt.

Für die hier vorliegende Erhebung wurde das Debat-O-Meter als Druckknopf-System im Reset Mode implementiert, d. h. dass nur Werte übermittelt werden, wenn aktiv ein Knopf gedrückt wurde. Über eine graduelle Abstufung von „++“ für eine sehr gute bis hin zu „−−“ für eine sehr schlechte Bewertung konnten die Versuchsteilnehmer mit dem eigenen mobilen Endgerät ihren momentanen Eindruck über die Spitzenkandidaten rückmelden. Für die Auswertung werden die aktiven Eingaben auf eine Skala von +2 bis −2 rekodiert. Inaktivität, also das Nicht-Drücken eines Knopfes, wurde gemäß der Messanweisung als neutraler Eindruck interpretiert und entspricht folglich dem Wert 0.

### Setting

Die Erhebung wurde als Feldstudie durchgeführt und präsentiert folglich einen komplementären Ansatz zu den im Forschungsbereich dominierenden Strategien mit laborexperimentellen Studiendesigns. Das Debat-O-Meter erlaubt die Nutzung von jedem Ort aus, an dem eine stabile Internetverbindung besteht, z. B. auf dem Sofa vor dem heimischen Fernseher. Sein linear-modularer Aufbau und die informationstechnischen Kontrollinstanzen (z. B. Nutzermonitoring, Sicherheitsarchitektur) gewährleisten gleichzeitig ein Mindestmaß an Kontrolle und Standardisierung der Stimulus-Rezeption und stärken so die Validität des Messverfahrens.

Es bleibt anzuführen, dass zwei mögliche Ursachen im Vergleich zu herkömmlichen RTR-Messungen einen Einfluss auf unsere Ergebnisse nehmen können: einerseits die neue Messung mit einem mobilen Endgerät und andererseits die weniger kontrollierten Bedingungen jenseits des Labors.^1^ Wenngleich wir dieses Konfundierungsproblem nicht gänzlich ausräumen können, so konnten wir doch an anderer Stelle mithilfe eines kontrollierten, quasi-laborexperimentellen Studiendesigns bereits zeigen, dass die Eingaben für zwei randomisierte Gruppen, von denen eine mit einer virtualisierten Implementierung und die andere mit physischen Eingabegeräten ausgestattet war, stark miteinander korreliert waren und eine äquivalente Datenstruktur generierten (vgl. Metz et al. 2016). Wir sind daher – in Anerkennung dieser Einschränkung – zuversichtlich, mithilfe unseres Designs in einer Feldstudie fundierte Aussagen über die Validität und Reliabilität von virtualisierten RTR-Messungen, die außerhalb des Labors implementiert werden, treffen zu können. Dies auch, weil wir mit unserer Applikation der Phasenstruktur klassischer RTR-Studiendesigns weitgehend folgen.

### Stimuli und Stichprobe

Der Beitrag untersucht exemplarisch Echtzeitdaten, die in Feldstudien zu TV-Duellen von drei Landtagswahlen im Wahljahr 2017 erhoben wurden: In Schleswig-Holstein duellierten sich Amtsinhaber Torsten Albig (SPD) und sein Herausforderer Daniel Günther (CDU), in Nordrhein-Westfalen fand das Duell zwischen der damaligen Ministerpräsidentin Hannelore Kraft (SPD) und ihrem späteren Nachfolger Armin Laschet (CDU) statt, während in Niedersachsen Ministerpräsident Stephan Weil (SPD) sein Amt gegen den CDU-Herausforderer Bernd Althusmann im verbalen Schlagabtausch zu verteidigen suchte.

Die Rekrutierung von Probanden unserer Studie erfolgte im Kern über eine breit angelegte Kooperation mit Print- und Online-Medien (u. a. Redaktionsnetzwerk Deutschland), die über das Projekt informierten und die Leserschaft zur Teilnahme aufriefen. Die Teilnahme war freiwillig, die Probanden erhielten keine geldwerte oder ähnliche Kompensation. Die im folgenden Abschnitt präsentierten Ergebnisse beruhen demnach auf einem *convenience sample*, was bedeutet, dass keine Zufallsauswahl aus einer Gesamtpopulation erfolgte. Wenngleich Ansätze des sogenannten *non-probability sampling* im Bereich der empirischen Debattenforschung in Deutschland etabliert sind (vgl. Maurer et al. 2007; Faas et al. 2017; Maier et al. 2014), so gehen mit der Wahl dieses Verfahrens merkliche Einschränkungen einher, die insbesondere in Prozessen der Selbstselektion gründen. Dies kann zu Verzerrungen in der Stichprobe führen, die wir auch in unserem Sample deutlich erkennen (siehe Tab. 1): So sind Männer in allen drei Samples deutlich überrepräsentiert, ebenso wie Personen mit einem ausgeprägten politischen Interesse und hohem formalem Bildungsgrad. Mit Blick auf die Altersstruktur zeigen sich spezifische Unterschiede: Während die Verteilung in Schleswig-Holstein beinahe ausgeglichen ist, ist das Sample in NRW vergleichsweise jung, wohingegen es in Niedersachsen von älteren Probanden dominiert ist. Das Verhältnis von Anhängern der Regierung und Opposition ist im Zweiküstenland weitgehend ausgeglichen. In NRW dominieren hingegen Anhänger der Opposition, während in Niedersachsen das Regierungslager das Studiensample prägt. Auch für die Verteilung von Personen ohne Parteiidentifikation zeigen sich deutliche Unterschiede zwischen den einzelnen Stichproben.

**Table 1:** 

	Schleswig-Holstein	Nordrhein-Westfalen	Niedersachsen
	(*N* = 269)	(*N* = 262)	(*N* = 173)
*Geschlecht*			
Weiblich	30,3	26,0	27,9
Männlich	69,7	74,0	72,1
*Alter*			
Bis 39 Jahre	47,9	64,7	37,3
40 Jahre und älter	52,1	35,3	62,7
*Bildung*			
Bis Mittlere Reife	19,5	16,4	24,2
Ab Fachhochschulreife	80,5	83,6	75,8
*Politikinteresse*			
Gering	24,2	14,7	13,4
Hoch	75,7	85,3	86,6
*Parteiidentifikation*			
AfD	0,4	1,1	1,8
Bündnis90/Die Grünen	6,4	4,6	11,2
CDU	30,5	54,8	27,8
Die LINKE	2,3	1,1	8,3
FDP	8,3	4,6	4,7
SPD	25,2	16,1	37,3
Andere	2,6	0,8	0,6
Keine	24,4	16,5	8,3

Angaben in Prozent

Vor diesem Hintergrund kann es nicht Ziel der Analyse sein, repräsentative Inferenzen auf eine wie auch immer geartete Grundgesamtheit abzuleiten. Vielmehr zielen wir darauf, die Beziehungen zwischen verschiedenen Variablen zu bewerten (vgl. Boydstun et al. 2014, S. 829–830). Des Weiteren beruhen die folgenden Untersuchungen in weiten Teilen auf Subgruppen-Analysen und individuellen Zeitreihen, was die Problematik von Verzerrungen deutlich abmildert. Deshalb haben wir bewusst auf jede Form der Gewichtung verzichtet, auch um den Eindruck zu verhindern, auf Grundlage unserer Stichprobe repräsentative Ergebnisse vorlegen zu wollen. Gleichzeitig gilt es, die Charakteristika der Stichprobe und die damit einhergehenden Einschränkungen bei der folgenden Interpretation unserer Ergebnisse zu berücksichtigen.

## Ergebnisse

### Konstrukt- und Kriteriumsvalidität

Um die Validität unserer virtualisierten RTR-Messung zu prüfen, nutzen wir drei Strategien. Die erste Analyseperspektive verfolgt mit dem Konzept der Konstruktvalidität die Frage, inwiefern unser Messkonzept dem zugrundeliegenden theoretischen Konstrukt entspricht (vgl. Wagschal 1999, S. 40). Bezogen auf die Perzeption politischer TV-Duelle folgen wir einem etablierten Ansatz in der empirischen Debattenforschung (vgl. Maier et al. 2007; Reinemann et al. 2005) und untersuchen, inwieweit das RTR-Signal mit den Parteibindungen der Studienteilnehmer assoziiert ist: Je besser das RTR-Signal durch die Parteiidentifikation vorhergesagt wird, desto stärker verhält es sich auf theoretisch plausible Weise und desto valider ist das RTR-Signal. In Hypothese 1 haben wir vor diesem Hintergrund die Parteiidentifikation als soziale Identität konzeptualisiert (vgl. Green et al. 2002). Damit ist eine wirkungsmächtige Freund-Feind-Logik für die Perzeption und Verarbeitung politischer Informationen grundlegend. Unter Bezugnahme auf konsistenztheoretische Überlegungen (vgl. Festinger 1962; Heider 1958; Faas und Maier 2004b), die die selektive Wahrnehmung politischer Inhalt befördern, gehen wir davon aus, dass die verschiedenen parteipolitischen Anhängergruppen in ihren Echtzeitbewertungen signifikant voneinander unterscheidbar sind (H1). Um diese Annahme zu prüfen, implementieren wir erstens Kruskal-Wallis-Tests, die anhand gruppenweiser Mittelwertvergleiche angeben, ob sich die Echtzeitbewertungen signifikant voneinander unterscheiden. Wir konzentrieren unsere Analyse dabei auf das Verhältnis der Anhängergruppen der in den TV-Duellen vertretenen Kandidaten. Dies zum einen deshalb, weil nur auf diese Untergruppen das Konzept der Parteiidentifikation als soziale Identität direkt übertragbar erscheint. Zum zweiten, weil die Teilnehmergruppen der nicht direkt in der Debatte vertretenen Parteien mitunter verhältnismäßig klein sind, weshalb die Unterschiede sehr groß sein müssten, um signifikant zu werden. Unter diesen Rahmenbedingungen zeichnet unsere Analyse für alle drei Duelle ein weitgehend einheitliches Bild: Die Anhänger der Christdemokraten unterscheiden sich von denjenigen der SPD in ihren Echtzeitbewertungen für die jeweiligen Kandidaten in signifikanter Weise.^2^

Als zweiten Ansatz zur Prüfung von H1 implementieren wir mit der linearen Diskriminanzanalyse (LDA) ein multivariates Verfahren zur Analyse von Gruppenunterschieden, mit dessen Hilfe wir unsere Einschätzung darüber verfeinern wollen, inwiefern die RTR-Eingaben zwischen den beiden Anhängergruppen der Diskussionsteilnehmer verschieden sind. Grundgedanke dieses Verfahrens ist es zu untersuchen, wie sich Gruppen (hier: Parteianhänger) jenseits einfacher Mittelwertvergleiche durch bestimmte Merkmale (hier: individuelle RTR-Bewertungen) unterscheiden lassen. Für dieses Vorhaben wurden die Eingaben eines jeden Teilnehmers für jeden Diskutanten saldiert. Anschließend wurden die Werte z‑standardisiert und die oberen und unteren 2,5 % der am extremsten Bewertenden aus dem Datensatz entfernt, um den Einfluss von Ausreißern zu reduzieren. Daraufhin wurde der Datensatz auf Anhänger von SPD und CDU zugeschnitten und jeweils in einen Trainings- und Testdatensatz zufällig halbiert. Auf Grundlage des Trainingsdatensatzes wurde eine Diskriminanzfunktion geschätzt. Mit Hilfe dieser Diskriminanzfunktion wurden sodann die Elemente der Teststichprobe klassifiziert und hierfür die bereinigte Trefferquote berechnet. Dieser Vergleich der – durch die Diskriminanzfunktion bewirkten – Klassifizierung der Untersuchungsobjekte mit deren tatsächlicher Gruppenzugehörigkeit ermöglicht es, die Güte (Trennkraft) der Diskriminanzfunktion zu beurteilen.

Wie aus Tab. 2 ersichtlich, ist die bereinigte Trefferquote für alle Duelle beachtlich: Sie beträgt in Schleswig-Holstein 79 %, in Nordrhein-Westfalen 89 % und in Niedersachsen 85 %. Allerdings unterscheiden sie sich deutlich zwischen den Anhängergruppen, insbesondere in Schleswig-Holstein und Nordrhein-Westfalen, für welche die Diskriminanzfunktion CDU-Anhänger sehr viel effizienter klassifiziert. Insgesamt sind wir bei dieser Befundlage zuversichtlich, Hypothese 1 – unter der Einschränkung der Gültigkeit auf die beiden im Duell repräsentierten Anhängergruppen – annehmen zu können.

**Table 2:** 

Tatsächliche Gruppenzugehörigkeit	Prognostizierte Gruppenzugehörigkeit	
	CDU	SPD
*Schleswig-Holstein*		
CDU	35 (90 %)	4 (10 %)
SPD	11 (33 %)	22 (67 %)
*Nordrhein-Westfalen*		
CDU	69 (97 %)	2 (3 %)
SPD	8 (40 %)	12 (60 %)
*Niedersachsen*		
CDU	18 (78 %)	5 (22 %)
SPD	3 (10 %)	28 (90 %)

Um zu einer weiteren Klärung der Befundlage beizutragen, rekurrieren wir auf ein etabliertes Verfahren zur Beurteilung der Validität von RTR-Messungen und implementieren die Methode der Strukturgleichungsmodellierung (vgl. Maier 2007b; Maier et al. 2007, 2014, 2016). Wir modellieren hierbei zwei verschiedene Strukturgleichungen: Ein schlankes Modell (vgl. Maier et al. 2016, S. 550–551) gibt einerseits Hinweise auf die statistischen Beziehungen zwischen der Parteiidentifikation und der Echtzeitbewertung und kann so die Befunde zur Konstruktvalidität ergänzen. Kern des Modells ist allerdings die Beurteilung der Assoziation zwischen RTR-Ratings und der retrospektiven Wahrnehmung der Debattenleistung bei Kontrolle des Einflusses der Parteiidentifikation. Unter der Annahme, dass die unmittelbaren Wahrnehmungen während der Debatte zu einem Gesamturteil über die Debattenleistung nach der Diskussion saldieren, lässt dieses schlanke Modell fundierte Aussagen über die Kriteriumsvalidität der virtualisierten RTR-Messung zu. Die Parteiidentifikation ist dabei als Dummy-Variable operationalisiert, das RTR-Rating als durchschnittliche Echtzeitbewertung während der gesamten Debatte und das retrospektive Urteil über die Debattenleistung als Bewertung des Kandidaten nach der Diskussion auf einer Fünfer-Skala.^3^ Die Ergebnisse sind in Tab. 3 abgebildet.

**Table 3:** 

AV	UV	Torsten Albig (SPD)	Daniel Günther (CDU)	Hannelore Kraft (SPD)	Armin Laschet (CDU)	Stephan Weil (SPD)	Bernd Althusmann (CDU)
RTR	Parteiidentifikation	0,343***	0,486***	0,633***	0,466***	0,539***	0,574***
Debattenleistung	Parteiidentifikation	−0,015	0,025	0,055	−0,003	−0,005	0,008
	RTR	0,894***	0,854***	0,794***	0,801***	0,862***	0,880***
RTR R^2^	–	0,118	0,236	0,401	0,217	0,291	0,330
Debattenleistung R^2^	–	0,791	0,750	0,688	0,640	0,739	0,782
χ^2^	–	381,509	374,135	407,210	308,129	282,326	323,228
*p*	–	0,000	0,000	0,000	0,000	0,000	0,000
RMSEA	–	0,000	0,000	0,000	0,000	0,000	0,000
SRMR	–	0,000	0,000	0,000	0,000	0,000	0,000
CFI	–	1,000	1,000	1,000	1,000	1,000	1,000
*N*	–	242	242	246	246	168	168

*df* = 1

***p < 0,01; **p < 0,05; *p < 0,10

Bei der globalen Betrachtung von Tab. 3 kann erstens festgehalten werden, dass alle Modelle bei den globalen Fit-Indices (aber nicht beim einem Chi^2^-Test) eine gute Anpassung an die Daten aufweisen. Zweitens sind die Ergebnisse der Strukturgleichungen mit Blick auf die Konstruktvalidität sehr konsistent: Parteiidentifikation und Echtzeitbewertung sind hochsignifikant und substanziell miteinander assoziiert. Dies bestätigt die bisherigen Befunde über die Diskriminierungsfähigkeit der Parteiidentifikation für Anhängergruppen der beiden Hauptprotagonisten in den jeweiligen Duellen und verweist auf die Konstruktvalidität der virtualisierten RTR-Messung (vgl. Hypothese 1). Allerdings unterscheidet sich die Varianzreduktion zwischen den Kandidaten und Duellen deutlich, was wir als Hinweis darauf lesen, dass die Parteiidentifikation je nach Kontext und Bewertungsobjekt (Kandidat) die Wahrnehmung der Kandidatenaussagen unterschiedlich stark filtert. Drittens zeichnen unsere Modelle mit Blick auf die Kriteriumsvalidität ein positives und einheitliches Bild: Das retrospektive Urteil über die Debattenleistung kann – unter Kontrolle der Parteiidentifikation – maßgeblich als Funktion der Echtzeitevaluationen gelten. Die standardisierten Koeffizienten sind sehr stark ausgeprägt und statistisch signifikant. Viertens zeigen alle Modelle eine hohe Erklärungskraft. Die Varianzreduktion liegt bei 64 bis 79 %, unterscheidet sich aber zwischen den Duellen deutlich: Während die Modelle für die Debatte in Schleswig-Holstein (Albig/Günther) und Niedersachsen (Weil/Althusmann) auf einem ähnlich hohen Niveau liegen, performieren die Strukturgleichungen für Kraft und Laschet in NRW insgesamt schwächer. Dies kann als Hinweis gelesen werden, dass die vorliegende Struktur grundsätzlich konsistent ist und eine bemerkenswerte Erklärungskraft entfaltet, gleichzeitig aber auch auf die Spezifika und unterschiedlichen Kontexte der einzelnen Duelle sensibel reagiert. Insgesamt finden wir also starke Belege für Hypothese 2 und die Kriteriumsvalidität unserer virtualisierten RTR-Messungen: Die Echtzeit-Evaluationen und retrospektiven Urteile über die Debattenleistung sind unter Kontrolle der politischen Voreinstellung der Parteiidentifikation signifikant und substanziell assoziiert.

### Inhaltsvalidität

Mit der Inhaltsvalidität prüfen wir, inwiefern unser Messinstrument sachlich und logisch in der Lage ist, das interessierende Merkmal (RTR-Signal) zu erfassen (vgl. Wagschal 1999, S. 40). Demnach gehen wir davon aus, dass die virtualisierte RTR-Messung eine Struktur der Debattenrezeption abbildet, wie sie aus Untersuchungen mit laborexperimentellen Studiendesigns und physischen Eingabegeräten (vgl. Bachl 2013; Maier 2007b) bekannt ist. Hierbei nehmen wir in Hypothese 3 an, dass die unmittelbaren Wahrnehmungen in Echtzeit substanziellen Einfluss auf die retrospektiven Kandidatenbewertungen haben (H3). Um unsere Erwartungen zu begründen, rekurrieren wir auf ein Strukturgleichungsmodell von Bachl (2013, S. 172), welches das RTR-Signal in ein komplexes Beziehungsgeflecht vorgelagerter und nachgelagerter Variablen der Debattenrezeption einbettet: Die unmittelbaren Wahrnehmungen (wie sie durch das RTR-Signal erfasst werden) gelten als maßgeblich von vorherigen Kandidatenbewertungen und Leistungserwartungen vorgeformt. Diese drei Variablen wiederum prägen die retrospektiven Urteile über die Debattenleistung, wohingegen die retrospektive Kandidatenbewertung nach der Debatte als Funktion der vier vorgenannten Variablen der Debattenrezeption gilt. Wir haben die Pfadstruktur aus Bachls Modell mit unseren Daten reproduziert und versucht, seiner Operationalisierung so nah wie möglich zu folgen (siehe Bachl 2013, S. 177 für eine ausführliche Darstellung seiner Operationalisierung sowie Anhang B für die Operationalisierung in unserer Studie). Die Ergebnisse sind in Tab. 4 dargestellt.

**Table 4:** 

AV	UV	Torsten Albig (SPD)	Daniel Günther (CDU)	Hannelore Kraft (SPD)	Armin Laschet (CDU)	Stephan Weil (SPD)	Bernd Althusmann (CDU)
Erwartete Debattenleistung	Kandidatenbewertung (vor)	0,661***	0,693***	0,712***	0,792***	0,732***	0,683***
Echtzeitbewertung (RTR)	Kandidatenbewertung (vor)	0,540***	0,635***	0,583***	0,641***	0,534***	0,543***
	Erwartete Debattenleistung	0,113*	0,000	0,116*	−0,025	0,245***	0,235***
Wahrgenommene Debattenleistung	Kandidatenbewertung (vor)	−0,031	0,043	−0,027	0,032	0,055	0,102**
	Erwartete Debattenleistung	0,058	0,015	0,223***	0,033	−0,060	−0,055
	Echtzeitbewertung (RTR)	0,873***	0,826***	0,743***	0,767***	0,857***	0,842***
Kandidatenbewertung (nach)	Kandidatenbewertung (vor)	0,114***	0,113***	0,218***	0,198***	0,084	0,170**
	Echtzeitbewertung (RTR)	0,357***	0,324***	0,354***	0,363***	0,761***	0,489***
	Wahrgenommene Debattenleistung	0,491***	0,529***	0,401***	0,394***	0,105	0,321***
Erwartete Debattenleistung R^2^	–	0,438	0,480	0,507	0,627	0,536	0,466
Echtzeitbewertung (RTR) R^2^	–	0,386	0,403	0,450	0,385	0,537	0,525
Wahrgenommene Debattenleistung R^2^	–	0,779	0,742	0,743	0,646	0,739	0,780
Kandidatenbewertung (nach) R^2^	–	0,805	0,808	0,785	0,728	0,836	0,837
χ^2^	–	0,692	1,998	0,162	0,001	0,704	4,065
*p*	–	0,405	0,158	0,687	0,971	0,401	0,044
RMSEA	–	0,000	0,077	0,000	0,000	0,000	0,154
SRMR	–	0,004	0,006	0,002	0,000	0,004	0,010
CFI	–	1,000	0,997	1,000	1,000	1,000	0,990
*N*	–	262	260	252	254	163	163

*df* = 1

***p < 0,01; **p < 0,05; *p < 0,10

Um die Gesamtgüte der vorliegenden Modelle zu beurteilen, stützen wir uns erneut auf die etablierten Fitmaße Chi-Quadrat, RMSEA, SRMR und CFI (vgl. Weiber und Mühlhaus 2014, S. 203–223). Das inferenzstatistische Gütekriterium RMSEA zeigt für fünf der sechs Modelle eine gute Modellanpassung, wohingegen die Maßzahl für Althusmann über dem empfohlenen Schwellenwert (≤0,10) für einen akzeptablen Modell-Fit liegt. Auch Chi-Quadrat zeigt dieses Muster (Schwellenwert ≤3). Prüfen wir für dieses Modell hingegen den absoluten Fit-Index SRMR, der die quadrierten Differenzen durch das Produkt der empirischen Varianzen der Variablen bereinigt, weist dieser Wert entsprechend dem Cutoff-Kriterium von ≤0,10, ebenso wie für alle anderen berechneten Strukturgleichungsmodelle, auf eine zufriedenstellende Modellanpassung hin. Diese positive Beurteilung der Anpassung unserer Strukturmodelle wird durch die Inspektion des inkrementellen CFI-Fitmaßes zum Modellvergleich bestätigt, da für alle Duelle und Kandidaten die Werte über dem Schwellenwert von ≥0,90 liegen. Darüber hinaus zeigen alle Modelle erneut eine hohe Erklärungskraft. Die Varianzreduktion liegt zwischen 72 % und 84 %, wobei das Modell für Laschet gegenüber den anderen deutlich schlechter performiert. Mit Blick auf die grundlegenden Strukturen der Debattenrezeption zeichnet Tab. 4 ein ziemlich einheitliches Bild: Die erwartete Debattenleistung ist maßgeblich von der Kandidatenbewertung vor der Diskussion determiniert. Deren Prägekraft ist auch für die Echtzeitbewertungen bestimmend. Die RTR-Evaluationen wiederum sind der bestimmende Faktor für die retrospektiven Urteile über die Debattenleistung – das komplexere Modell bestätigt also die zuvor getroffenen Befunde über die Kriteriumsvalidität. Etwas uneinheitlicher ist das Bild bei der Kandidatenevaluation nach der Debatte: Die vorherige Kandidatenbewertung ist in fünf Modellen ein signifikanter, wenngleich nicht substanzieller Faktor. Als substanziell kann hingegen die wahrgenommene Debattenleistung gelten, auch wenn diese Variable erneut für das Modell von Weil keinen Beitrag leistet. Umso größer ist in diesem Modell der Einfluss der Echtzeitwahrnehmungen auf die retrospektive Kandidatenbewertung. Und auch für alle anderen Modelle gilt, dass die RTR-Bewertungen deutlich mit den Kandidatenevaluationen nach der Debatte assoziiert sind. Wir finden also über alle Modelle hinweg Evidenz für die Beibehaltung unserer Hypothese 3, wonach die unmittelbaren Wahrnehmungen in Echtzeit substanziellen Einfluss auf die retrospektiven Kandidatenbewertungen nehmen (H3). Insgesamt können wir die empirischen Befunde von Bachl (2013, S. 183–186) mit unseren Berechnungen weitgehend replizieren. Ein leichter Unterschied hierzu ergibt sich mit Blick auf die etwas höhere Bedeutung der Echtzeitbewertung für die retrospektive Debattenleistung und Kandidatenbewertung in unseren Modellen. Allgemein zeichnen die hier berechneten Strukturgleichungsmodelle ein recht konsistentes Bild über den Rezeptionsprozess und die Beziehungsmuster der Echtzeitbewertung zu vor- und nachgelagerten Variablen der Debattenrezeption, was als deutlicher Hinweis auf die Inhaltsvalidität unserer virtualisierten RTR-Messung gelten kann.

### Reliabilität

**Figure 1:**
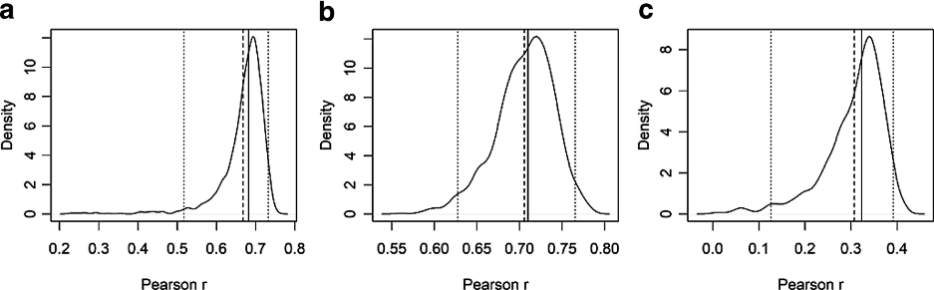

Um die Reliabilität virtualisierter RTR-Messverfahren zu beurteilen, verfolgen wir zwei Ansätze. Zum einen analysieren wir die *aggregierten* RTR-Zeitreihen, d. h. die aufsummierten RTR-Eingaben der Teilnehmer pro Zeiteinheit. Dabei folgen wir dem Vorgehen von Bachl (2014, S. 102–103) und implementieren ein Resampling-Verfahren. Wir gehen dafür in drei Schritten vor: Zunächst wird die Stichprobe nach dem Zufallsprinzip in zwei Teilstichproben mit gleichem Umfang aufgeteilt. In einem zweiten Schritt werden für beide Teilstichproben die aggregierten RTR-Zeitreihen über ein gleitendes Mittel von fünf Sekunden berechnet. Zuletzt wird die Korrelation (Pearsons r) zwischen den Zeitreihen ermittelt. Dieses Verfahren wird 1000-mal wiederholt, und die daraus resultierenden Korrelationen zwischen den RTR-Zeitreihen der Teilstichproben werden notiert. Aus den Verteilungen der Korrelationen des Resampling-Verfahrens können wir beurteilen, wie sehr der Verlauf der aggregierten RTR-Zeitreihen von der Zusammensetzung der Stichprobe abhängt. Unsere Ergebnisse sind in Abb. 1 dargestellt. In den Grafiken sind die Verteilungen der Korrelationen für die aggregierten Zeitreihen aller Studienteilnehmer über den gesamten Verlauf des TV-Duells hinweg eingezeichnet. Darüber hinaus sind zusätzlich der Median (durchgezogen), der Durchschnittswert (gestrichelt) sowie das 2,5- und 97,5-Perzentil (gepunktet) als vertikale Linien abgetragen. Der Bereich zwischen den gepunkteten Linien entspricht damit dem 95 %-Resampling-Konfidenzintervall der Korrelationen zwischen den aggregierten RTR-Zeitreihen aus zwei zufällig gebildeten Hälften der jeweiligen Stichproben.

In Hypothese 4 nehmen wir an, dass die Korrelationskoeffizienten im Resampling-Verfahren über dem Schwellenwert von 0,6 liegen (vgl. Wagschal 1999, S. 197), um eine akzeptable Reliabilität der aggregierten RTR-Zeitreihen zu indizieren.^4^ In Schleswig-Holstein liegen Median (0,68) und durchschnittlicher Korrelationskoeffizient (0,67) über dem Schwellenwert und weisen damit auf die Reliabilität der aggregierten RTR-Zeitreihen hin. Einschränkend ist allerdings anzuführen, dass das untere Konfidenzintervall (0,53) den Grenzwert von 0,6 (leicht) unterschreitet. Diese Feststellung gilt nicht für die aggregierten Echtzeitdaten in NRW; das 2,5-Perzentil liegt hier bei 0,63 und damit über dem kritischen Schwellenwert. Folglich liefern auch Median (0,71) und der Durchschnittswert (0,70) klare Hinweise auf die Reliabilität der RTR-Daten, die mithilfe des Debat-O-Meters in einer Feldstudie außerhalb des Labors erhoben wurden. Deutlich geringer fallen die Koeffizienten hingegen für das Duell in Niedersachsen aus. Nicht nur das untere Konfidenzintervall (0,14), sondern auch das arithmetische Mittel (0,30) und der Median (0,32) unterschreiten den Schwellenwert deutlich. Selbst das 97,5-Perzentil (0,39) und der Maximalwert (0,43) bleiben hinter der in Hypothese 4 formulierten Erwartung zurück. Die Ergebnisse ziehen damit die Reliabilität der Echtzeitdaten insbesondere aus der TV-Duell-Studie in Niedersachsen in Zweifel.^5^

Wie lassen sich die ambivalenten Befunde über die Korrelationskoeffizienten der aggregierten RTR-Zeitreihen im Resampling-Verfahren beurteilen? Eine erste mögliche Perspektive richtet sich auf das Eingabegerät. Die softwareseitige Implementierung war für alle drei Studien als Push-Button-System im Reset mode identisch. Allerdings haben wir in unserer Feldstudie keinen Einblick darüber, welche Hardware die Probanden nutzten (PC, Notebook, Tablet, Smartphone) und ob dies zu systematischen Unterschieden in den Eingaben führte. Zukünftige Untersuchungen könnten auf die verwendete Hardware kontrollieren. Eine zweite Perspektive ergibt sich hinsichtlich der Messanweisung. Diese war in allen drei Studien gleich ausgestaltet und ist den Anweisungen aus laborexperimentellen Studien entlehnt (vgl. Bachl 2014, S. 96), die von Probanden eine Rückmeldung über den allgemeinen Eindruck der Diskutierenden erfragen. Diese eher offene und im Vergleich zu Labormessungen erheblich gekürzte Formulierung über das zu erhebende Konstrukt könnte zu sehr unterschiedlichen inhaltlichen Interpretationen der Messanweisung geführt und zwischen den Teilnehmern Bewertungsdimensionen mit unterschiedlicher Ausprägung (z. B. Sympathie oder Kompetenz) befördert haben. Zukünftige Forschung sollte folglich einen systematischen Vergleich von Messanweisungen leisten, die in ihrer Spezifizierung der Bewertungsdimensionen variieren (vgl. Bachl 2014, S. 41). Eine dritte Perspektive lenkt den Blick auf die Bewertungsobjekte. Während die Rezipienten bei den Duellen in Schleswig-Holstein und Nordrhein-Westfalen ausschließlich die beiden Kandidaten bewerteten, wurden die Probanden in Niedersachsen zusätzlich gebeten, ihren aktuellen Eindruck zur Moderation mitzuteilen, d. h. dass die Anzahl der Bewertungsobjekte erhöht war bei gleichbleibender Ausprägung von Bewertungsitems (positiv vs. negativ) und -dimension (allgemeiner Eindruck) zwischen den Studien. Dies könnte als Hinweis gelesen werden, dass die Anzahl an Bewertungsobjekten Einfluss auf die Reliabilität der aggregierten RTR-Zeitreihen nimmt. Um dies weiter zu plausibilisieren, sollten zukünftige Forschungsarbeiten systematisch die Anzahl an Bewertungsobjekten variieren. Viertens ist der Medienstimulus selbst zu beachten. Unsere Studie kontrolliert nicht auf den Inhalt der einzelnen Debatten. Dabei ist bekannt, dass die inhaltliche Ausgestaltung eines Duells wie beispielsweise die rhetorischen Strategien der Diskutierenden entscheidend Einfluss auf die interindividuellen Bewertungsmuster nehmen können. Denn während Angriffe auf den politischen Gegner und konkrete Selbstpräsentationen politische Voreinstellungen aktivieren und so die unmittelbare Wahrnehmung zwischen politischen Anhängergruppen polarisieren, können Allgemeinplätze lagerübergreifend Zustimmung erfahren (vgl. Reinemann und Maurer 2005, 2007). All diese Aspekte könnten dazu beitragen, dass die aggregierten Zeitreihen in hohem Maße von der individuellen Zusammensetzung der Stichprobe abhängig sind, weshalb die Korrelationskoeffizienten im Resampling-Verfahren zwischen den Studien stark variieren. Dass das Bewertungsverhalten zwischen den Studien stark variiert, können wir auch an der deskriptiven Statistik ersehen. Während die 269 Probanden in Nordrhein-Westfalen 100.986 Bewertungen in 3723 s und damit durchschnittlich rund alle 10 s eine RTR-Eingabe abgaben, liegt dieser Wert für Schleswig-Holstein bei 16 s (56.294 RTR-Eingaben von 262 Nutzern in 3516 s) und für Niedersachsen bei 38 s (19,665 Inputs von 173 Personen in 4334 s). Die Aussagen der Kandidaten in Niedersachsen erzeugten nicht nur weniger Reaktionen, sondern scheinen sich zudem weniger systematisch im Zeitverlauf des Medienstimulus zu verteilen, was sich negativ auf die Korrelationskoeffizienten im Resampling-Verfahren auswirkt.

Vor diesem Hintergrund sind die hier präsentierten Ergebnisse ambivalent zu beurteilen. Einerseits zeigen sie, dass virtualisierte Messverfahren im Feld akzeptable Reliabilitätswerte der aggregierten Zeitreihen generieren können. Andererseits lassen die ambivalenten Ergebnisse – nicht nur zur Studie in Niedersachsen – Zweifel an der Reliabilität der aggregierten RTR-Zeitreihen aufkommen. Dies wird besonders deutlich, wenn wir bedenken, dass die gemeinsame Varianz (R^2^) der jeweiligen Halbgruppen in den Resampling-Verfahren in allen Studien im Durchschnitt bei unter 50 % verbleibt. Zudem wird deutlich, dass noch weitgehend Unkenntnis über die Faktoren herrscht, die die Reliabilitätswerte der aggregierten Zeitreihen systematisch beeinflussen. Wir lehnen deshalb Hypothese 4 ab.

Um die Reliabilität der *individuellen* RTR-Zeitreihen zu beurteilen, rekurrieren wir in der folgenden Analyse auf die etablierten Indikatoren McDonalds Omega und Cronbachs Alpha (vgl. Papastefanou 2013; Bachl 2014, S. 108–110; Waldvogel und Metz 2020). In Hypothese 5 haben wir die Annahme formuliert, dass die beiden Koeffizienten, die die interne Konsistenz der individuellen Zeitreihen messen, über dem kritischen Schwellenwert von 0,8 verbleiben sollten (H5). Um die Hypothese zu prüfen, haben wir die Debatten in die einzelnen Sprechphasen unterteilt.^6^ Diese dienen uns als Quasi-Items, um die Indikatoren der internen Konsistenz zu berechnen. Für jede Sprechphase haben wir die positiven und negativen Evaluationen, die ein Teilnehmer innerhalb eines bestimmten Intervalls für den jeweiligen Kandidaten abgegeben hat, separat aufsummiert. Auf Basis dieser vier Matrizen haben wir dann McDonalds Omega und Cronbachs Alpha berechnet. Wir stützen unsere Analyse auf diese beiden Indikatoren der internen Konsistenz aus zwei wesentlichen Gründen. Erstens ist Cronbachs Alpha als Indikator zwar weit verbreitet, aber mit dem Problem behaftet, dass der Koeffizient in erheblichem Maße von der Anzahl der Items abhängt. Für eine hohe Itemzahl kann selbst eine relativ niedrige Interkorrelation zwischen den Sprechphasen den Indikator auf relativ hohe Werte hochtreiben, wodurch möglicherweise ein zu optimistischer Eindruck über die Reliabilität entsteht. Konkret bedeutet dies für unsere Beispiele, dass bei einer Itemzahl von 42 Sprechphasen (Kraft) bereits eine Korrelation von 0,087 den Koeffizienten auf den Schwellenwert von 0,8 heben würde, wohingegen bei 16 Sprechphasen (Günther) die Korrelation 0,201 betragen müsste. Für McDonalds Omega besteht diese Abhängigkeit zur Itemzahl hingegen nicht. Zweitens wurde Cronbachs Alpha für die Annahme kritisiert, dass alle Items ähnlich auf das gemessene Konstrukt laden (vgl. Dunn et al. 2014). Omega hingegen teilt diese Annahme nicht, so dass die Ladungen der einzelnen Items variieren können (vgl. Kelley und Pornprasertmanit 2016; siehe Zinbarg et al. 2005 sowie Revelle und Zinbarg 2009 für einen Vergleich von Alpha und Omega). Man könnte daher argumentieren, dass die Fokussierung auf Omega hinreichend ist, um die Reliabilität der individuellen RTR-Zeitreihen zu beurteilen. Wir haben uns jedoch dazu entschieden, auch Alpha zu berechnen (einschließlich seiner Bootstrap-Konfidenzintervalle, wie in Padilla et al. 2012 beschrieben), da eine interessante Implikation, die den verschiedenen Messmodellen zugrunde liegt, darin besteht, dass Omega gegenüber Alpha substanziell höher ausfallen sollte, wenn die Faktorladungen tatsächlich über die Zeit variieren. Da unseren Items ein und dieselbe Messanweisung zugrunde liegt, würden über die Zeit variierende Ladungen signalisieren, dass die Teilnehmer sich der Messanweisung manchmal mehr oder weniger bewusst sind. Folglich gibt jeder systematische Unterschied zwischen Alpha und Omega Aufschluss darüber, wie sehr die Teilnehmer die Messanweisung während der Debatte befolgt haben (Waldvogel und Metz 2020, S. 666).

In der globalen Betrachtung der in Tab. 5 dargestellten Ergebnisse zeigt sich, dass beide Koeffizienten eine hohe interne Konsistenz der Datenreihen belegen. In Schleswig-Holstein liegt Omega zwischen 0,973 und 0,931, für das NRW-Duell zwischen 0,992 und 0,937 und beim Duell in Niedersachsen zwischen 0,972 und 0,932. Diese insgesamt sehr positive Einschätzung der internen Konsistenz der virtualisierten RTR-Daten wird durch Alpha bestätigt. Der höchste Wert beträgt 0,959 für positive Echtzeitevaluationen für Daniel Günther im Zweiküstenland. Und selbst der niedrigste Wert von 0,904 für negative RTR-Eingaben für Armin Laschet im NRW-Duell bleibt klar über der kritischen Schwelle von 0,8. Damit zeigen sich deutliche Belege, die in Einklang mit Hypothese fünf (H5) stehen: Die individuellen Zeitreihen der RTR-Daten weisen eine hohe interne Konsistent auf. Allerdings ist zu diesem Befund anzumerken, dass die hohen Werte der internen Konsistenz unserer RTR-Daten nicht uneingeschränkt positiv zu beurteilen sind. Denn wenn wir bedenken, dass die RTR-Messungen während eines sich verändernden Stimulus erfasst werden, könnte die hohe Konsistenz auch als ein Zeichen dafür interpretiert werden, dass die individuellen RTR-Messungen nur wenig sensibel auf Veränderungen des Stimulus reagieren (vgl. Bachl 2014, S. 61).

**Table 5:** 

	McDonald’s Omega		Cronbach’s Alpha	
	Positiv	Negativ	Positiv	Negativ
Torsten Albig	0,944 (0,935; 0,954)	0,973 (0,969; 0,978)	0,919 (0,874; 0,964)	0,946 (0,935; 0,957)
Daniel Günther	0,974 (0,969; 0,978)	0,931 (0,919; 0,943)	0,959 (0,947; 0,971)	0,911 (0,883; 0,939)
Hannelore Kraft	0,992 (0,991; 0,994)	0,965 (0,958; 0,971)	0,948 (0,896; 1,000)	0,948 (0,939; 0,958)
Armin Laschet	0,969 (0,963; 0,974)	0,937 (0,890; 0,983)	0,953 (0,946; 0,960)	0,904 (0,867; 0,941)
Stephan Weil	0,972 (0,966; 0,978)	0,954 (0,944; 0,964)	0,957 (0,944; 0,970)	0,939 (0,912; 0,966)
Bernd Althusmann	0,932 (0,917; 0,946)	0,937 (0,924; 0,951)	0,924 (0,903; 0,944)	0,935 (0,902; 0,968)

Ungeachtet dessen können wir feststellen, dass Omega zwar durchgehend leicht höhere Koeffizienten aufweist als Alpha, beide Indikatoren aber hoch miteinander korreliert sind. Die Unterschiede zwischen den beiden Parametern scheinen deshalb insgesamt nicht substanziell, was darauf hindeutet, dass die Faktorladungen tatsächlich relativ konstant sind. Dies kann als Beleg dafür interpretiert werden, dass unsere Studienteilnehmer die Messanweisungen im Verlauf der Debatte allgemein befolgt haben. Dieser Befund ist vor dem Hintergrund der geringeren Standardisierung und Kontrolle der Rezeptionssituation in RTR-Feldstudien positiv zu bewerten, weil er eine adäquate Anwendung der Messmethode durch die Studienteilnehmer auch außerhalb des Labors indiziert.

## Diskussion

In diesem Beitrag haben wir das Ziel verfolgt, die Validität und Reliabilität von virtualisierten RTR-Messungen zu drei TV-Duellen vor Landtagswahlen zu beurteilen. Hierbei konnten Zuschauer einer TV-Debatte ihre unmittelbaren Wahrnehmungen mithilfe einer RTR-Applikation mit dem eigenen mobilen Endgerät in natürlichen Rezeptionssituationen sekundengenau rückmelden. Mit dem Verlassen des Labors geht eine erheblich geringere Standardisierung und Kontrollmöglichkeit der Stimulusrezeption einher.

Wir haben deshalb in unserer ersten Forschungsfrage die Validität der RTR-Daten in dreifacher Weise untersucht. Zuerst haben wir die Konstruktvalidität betrachtet. Die Ergebnisse unserer Kruskal-Wallis-Tests zeigen eine statistisch signifikante Differenzierung des RTR-Bewertungsverhaltens entlang von Parteibindungen für Anhänger der beiden in den Duellen repräsentierten Parteien SPD und CDU. Diese positive Einschätzung wird durch die hohe Trefferquote der Diskriminanzanalyse unterstrichen. In einem weiteren Analyseschritt haben wir ein schlankes Strukturgleichungsmodell implementiert, das die Beziehung von Parteiidentifikation und RTR-Messung beurteilt und damit Aussagen über die Konstruktvalidität der virtualisierten RTR-Messung zulässt. Die errechneten Koeffizienten belegen, dass Parteiidentifikation und Echtzeitbewertung signifikant und substanziell miteinander assoziiert sind. Wenngleich sich unsere Analyse auf die beiden in den TV-Duellen direkt repräsentierten Parteianhänger konzentriert, so sehen wir doch in der Zusammenfassung der Ergebnisse die Frage nach der Konstruktvalidität (FF1.1) positiv beschieden und für unsere virtualisierten RTR-Daten gegeben.

Als zweite Perspektive haben wir die Untersuchung der Kriteriumsvalidität eröffnet. Ein zentraler Befund ergibt sich dabei aus der Betrachtung des schlanken Strukturgleichungsmodells, in dessen Kern die Beziehung von RTR-Rating und den retrospektiven Urteilen über die Debattenleistung steht. Unter Kontrolle der Parteiidentifikation konnten wir zeigen, dass die Koeffizienten sehr stark ausgeprägt und hochsignifikant sind. Diese Beobachtung gilt über alle Duelle und Kandidaten hinweg. Wir finden also ein sehr einheitliches Bild, was uns dazu veranlasst, die Frage nach der Kriteriumsvalidität (FF1.2) unserer virtualisierten RTR-Messung zu bejahen.

Als dritte Analyseperspektive haben wir die Inhaltsvalidität in den Blick genommen. Wir rekurrierten hierfür auf ein etabliertes Strukturgleichungsmodell (vgl. Bachl 2013, S. 172), das das RTR-Signal in ein komplexes Beziehungsgeflecht vorgelagerter und nachgelagerter Variablen der Debattenrezeption einbettet. Unsere Ergebnisse sind dabei weitgehend über alle Duelle und Kandidaten hinweg einheitlich: Die Echtzeitbewertungen während der Debatte sind durch politische Prädispositionen (vorherige Kandidatenbewertung) maßgeblich vorgeformt. Die wahrgenommene Debattenleistung ist – in Übereinstimmung mit unseren vorherigen Befunden – auch in diesem komplexeren Modell weitgehend eine Funktion der RTR-Ratings; dies unterstreicht die zuvor positive Beurteilung über die Kriteriumsvalidität der virtualisierten RTR-Messung. Die retrospektiven Kandidatenbewertungen wiederum sind maßgeblich von den Echtzeitbewertungen und den retrospektiven Urteilen über die Debattenleistung beeinflusst. Wir konnten mit diesem Ansatz folglich zeigen, dass die Struktur der Debattenperzeption in unseren Feldstudien zu Befunden, wie sie aus Untersuchungen in laborexperimentellen Settings mit physischen Eingabegeräten bekannt sind, korrespondieren (vgl. Bachl 2013, S. 183–186). Wir sind daher zuversichtlich, auch die Frage nach der Inhaltsvalidität (FF1.3) unserer virtualisierten RTR-Messung positiv beantworten zu können.

Mit unserer zweiten Forschungsfrage haben wir die Reliabilität der RTR-Daten ins Zentrum gerückt und in zweifacher Weise untersucht. Mithilfe eines Resampling-Verfahrens haben wir die aggregierten RTR-Zeitreihen in den Blick genommen. Einerseits zeigte sich, dass virtualisierte RTR-Messungen in der Tat Daten generieren können, deren aggregierte RTR-Zeitreihen für zwei Teilstichproben in einem Resampling-Verfahren deutlich miteinander assoziiert sind. Andererseits sind die Befunde ambivalent zu beurteilen, weil die bestehenden Korrelationen nur mittelstark und für das Niedersachsen-Duell gar nur gering ausgeprägt sind. Die RTR-Messung scheint also in nicht unerheblichem Maße abhängig von der Zusammensetzung der Stichprobe zu sein. Zukünftige Forschungsarbeiten sollten deshalb verstärkt Anstrengungen unternehmen, Quotierungsverfahren auf die Stichprobenrekrutierung auch im Online-Setting anzuwenden und gegebenenfalls stärker die Kandidaten(‑strategien) und ihre Aussagen in der Analyse zur Datenqualität zu berücksichtigen. In einer zweiten Analysestrategie haben wir die individuellen RTR-Zeitreihen auf ihre interne Konsistenz überprüft. Sowohl Cronbachs Alpha als auch McDonalds Omega belegen eine hohe Interkorrelation. Wenn wir unsere Befunde über die aggregierten und individuellen RTR-Zeitreihen zusammenfassen, so können wir unsere Forschungsfrage zwei (FF2) nur eingeschränkt positiv beantworten: Virtualisierte RTR-Messungen können reliable Daten generieren. Dies ist aber kein Automatismus; vielmehr müssen die Forscher durch besondere Sorgfalt die Voraussetzungen für ein adäquates Studiendesign auch im Online-Setting gewährleisten.

Unsere Ergebnisse unterliegen darüber hinaus Einschränkungen in mehrfacher Hinsicht: Zuvorderst ist anzumerken, dass unsere Untersuchung die eingangs formulierten Forschungsfragen beantworten möchte, indem sie methodologische Aspekte und Daten empirischer Studien zu drei Landtagswahlen im Jahr 2017 auskoppelt. Explizite Methodenstudien könnten demgegenüber systematischere Manipulationen der Rezeption ermöglichen, um weiterführende Erkenntnisse über etwaige Messartefakte, die Reichweite und Begrenzung dieser hochreaktiven Messmethode in seiner virtualisierten Form zu befördern. Des Weiteren ist anzumerken, dass mit dem Heraustreten aus dem Labor vielfältige Erwartungen bezüglich einer verbesserten externen Validität der getroffenen Befunde verknüpft sind. Allerdings kann festgehalten werden, dass Nachweise hierfür bisher in der methodologischen Forschungsliteratur fehlen. Vielmehr scheint es, dass sowohl laborexperimentell als auch virtualisiert erhobene RTR-Daten eine hinreichende Qualität aufweisen und einen je eigenen Beitrag zur empirischen Debattenforschung leisten können. Eine weitere Herausforderung (wenngleich nicht spezifisch für die virtualisierte Spielart der RTR-Datenerhebung) stellt das *convenience sample* dar. Während für Untergruppen-Analysen die Auswirkungen weniger stark ausgeprägt sein dürften, so können Prozesse der Selbstselektion zu starken Verzerrungen in der Gesamtstichprobe führen, was die Interpretationen über alle Teilnehmer hinweg erschwert. Auch wenn die (meist auf laborexperimentellen Settings mit physischen Eingabegeräten) bestehende Literatur bereits eine hohe Sensibilität dafür zeigt, nur eingeschränkt allgemeine Inferenzen auf eine Gesamtpopulation aus den eigenen Befunden abzuleiten, sind unsere Studien ein Hinweis dafür, dass diese Zurückhaltung auch für Feldstudien angezeigt ist, die ihre Daten virtualisiert in natürlichen Rezeptionssituationen erheben. Adäquate Formen der Gewichtung und Post-Stratifizierung könnten die skizzierten Probleme abmildern, allerdings liegen bisher keine Erkenntnisse über mögliche Techniken und ihre Anwendung auf die Erhebung von Echtzeitdaten vor. Ein weiteres Feld ist die Verbesserung der Standardisierung der Stimulusrezeption bei der onlinegestützten Erhebung rezeptionsbegleitend gemessener Zuschauerreaktionen. Denn die gemeinsame Rezeption und Interaktion mit anderen Personen in natürlichen Situationen kann das Individuum als grundlegende Untersuchungseinheit in Zweifel ziehen. Die Sortierung nach IP-Adressen, die explizite Aufforderung, während der Rezeption nicht zu interagieren oder gar allein zu schauen, könnten Strategien sein, um die Problematik einzuhegen. Viertens sollten sich zukünftige Forschungsarbeiten dem Konfundierungsproblem von neuer Messung und veränderten Messbedingungen widmen, um diese unterschiedlichen Einflussfaktoren auf die Messergebnisse zu entwirren.

Insgesamt zeichnen unsere getroffenen Befunde (unter Berücksichtigung der skizzierten Einschränkungen) ein positives Bild über die Qualität von RTR-Daten, die mithilfe eines virtualisierten Messinstrumentariums in Feldstudien erhoben wurden, in denen Zuschauer einer politischen TV-Debatte ihre unmittelbaren Eindrücke sekundengenau in natürlichen Rezeptionssituationen mit dem eigenen mobilen Endgerät rückmelden können. Dennoch steht die methodologische Forschung zu dieser neuartigen Methode der RTR-Datenerhebung erst am Anfang, weshalb weitere Untersuchungen angezeigt scheinen, um die Potenziale, aber auch die Limitationen virtualisierter RTR-Messung in Zukunft besser und detaillierter beurteilen zu können. Aus unseren Befunden können wir schließen, dass die Virtualisierung des RTR-Messinstrumentariums einen komplementären Ansatz zu den dominierenden Erhebungen mit laborexperimentellem Forschungsdesign etabliert, wodurch die Analyse von Publikumsreaktionen auch jenseits der TV-Duellforschung in natürlichen Rezeptionssituationen zugänglich wird. Dieser neue Ansatz gewährleistet zudem die technische Implementierung von RTR-Studien auch in Zeiten der COVID-19-Pandemie, in der klassische Experimentalstudien in Laborsettings mit physischen Eingabegeräten kaum durchführbar sind. Darüber hinaus erschließen sich neue Anwendungsszenarien, die durch die softwareseitige Implementierung sehr leicht die Untersuchung zum Beispiel von Mehrpersonen-Panels bzw. (mit Blick auf die Bundestagswahl 2021) eines Triells erlauben, aber auch methodische Erweiterungen, beispielsweise die Kombination mit Echtzeitdaten aus der Emotions- bzw. Gesichtserkennungssoftware, ermöglichen (vgl. Fridkin et al. 2021).

## Anhang B

**Table 6:**

Konstrukt (Befragungszeitpunkt)	Fragewortlaut	Antwortkategorien
Parteiidentifikation (vor dem Duell)	In Deutschland neigen viele Leute längere Zeit einer bestimmten politischen Partei zu, obwohl sie auch ab und zu eine andere Partei wählen. Wie ist das bei Ihnen: Neigen Sie – ganz allgemein gesprochen – einer bestimmten Partei zu? Und, wenn ja, welcher?	AfD^a^, Bündnis 90/Die Grünen^a^, CDU^a^, Die Linke^a^, FDP^a^, Piraten^a,b^, SPD^a^, SSW^a,b^, Einer anderen Partei, Nein, keiner Partei
Kandidatenbewertung (vor und nach dem Duell)	Was halten Sie ganz allgemein von den Spitzenkandidaten?	−2 = überhaupt nichts, 0 = teils/teils, +2 = sehr viel
Erwartete Debattenleistung (vor dem Duell)	Wie werden die einzelnen Spitzenkandidaten in der Diskussion jeweils abschneiden?	Sehr schlecht, Eher schlecht, Mittelmäßig, Eher gut, Sehr gut, Keine Angabe
Debattenleistung mittels RTR (während des Duells)	Mittelwert der individuellen RTR-Bewertungen eines jeden Teilnehmers	Graduelle Abstufung von „++“ für eine sehr gute bis hin zu „−−“ für eine sehr schlechte Bewertung
Wahrgenommene Debattenleistung (nach dem Duell)	Wie haben die einzelnen Spitzenkandidaten in der Diskussion jeweils abgeschnitten?	Sehr schlecht, Eher schlecht, Mittelmäßig, Eher gut, Sehr gut, Keine Angabe
Erwarteter Gewinner	Wer, glauben Sie, wird in der Diskussion am besten abschneiden?	<Kandidatennamen>^a^, Kein klarer Sieger
Wahrgenommener Gewinner	Alles in allem, wer hat Ihrer Meinung nach in der Diskussion am besten abgeschnitten?	<Kandidatennamen>^a^, Kein klarer Sieger

^a^Antwortkategorie im Fragebogen randomisiert

^b^Piraten nur in Nordrhein-Westfalen und Niedersachsen, SSW nur in Schleswig-Holstein

## Footnotes

1 Wir danken den unbekannten Gutachtern dafür, dies expliziert zu haben.

2 Schleswig-Holstein: Torsten Albig (Chi-Quadrat = 42,483, p = <0,001); Daniel Günther (38,736, p = <0,001); Nordrhein-Westfalen: Armin Laschet (Chi-Quadrat = 42,249, p = <0,001); Hannelore Kraft (Chi-Quadrat = 47,896, p = <0,001); Niedersachsen: Bernd Althusmann (Chi-Quadrat = 55,197, p = <0,001); Stefan Weil (Chi-Quadrat = 47,014, p = <0,001).

3 Da die Debattenleistung für das Duell in Niedersachsen nicht gesondert abgefragt wurde, ist diese für die Modelle Weil und Althusmann als ordinale Variable von −1 bis +1 für Niederlage, Unentschieden oder Sieg des jeweiligen Kandidaten operationalisiert.

4 Wir weisen erneut darauf hin, dass es keinen allgemeinen Schwellenwert für die Beurteilung von Korrelationen zwischen aggregierten RTR-Zeitreihen gibt (vgl. Maier et al. 2016) und dieser (willkürlich) gewählte Wert der allgemeinen Methodenliteratur (vgl. Wagschal 1999, S. 197) entlehnt ist.

5 Die Stichproben aller drei Studien sind stark von jungen, männlichen Teilnehmern geprägt. Unter Ausschluss dieser dominanten Gruppe stellen sich die durchschnittlichen Korrelationskoeffizienten wie folgt dar: Schleswig-Holstein = 0,64 (N = 81); Nordrhein-Westfalen = 0,51 (N = 68); Niedersachsen = 0,15 (N = 48).

6 Jede Sprechphase endete in unserer Berechnung fünf Sekunden nach dem Ende des eigentlichen Redebeitrags, um verzögerte RTR-Eingaben (sogenannte Latenzzeit) zu berücksichtigen (vgl. Nagel 2012, S. 152–157). Für die einzelnen Diskutanten ergaben sich folgende Sprechphasen: Albig: 19; Günther: 16; Kraft: 42; Laschet: 38; Weil; 39; Althusmann; 38.
